# Sporting programs for inactive population groups: factors influencing implementation in the organized sports setting

**DOI:** 10.1186/s13102-015-0007-8

**Published:** 2015-06-03

**Authors:** Linda Ooms, Cindy Veenhof, Nicolette Schipper-van Veldhoven, Dinny H. de Bakker

**Affiliations:** 1 Netherlands Institute for Health Services Research (NIVEL), PO Box 1568, 3500 BN Utrecht, The Netherlands; 2 Physical Therapy Research, Program in Clinical Health Sciences & Department of Rehabilitation, Nursing Science and Sport, Brain Center Rudolf Magnus, University Medical Center Utrecht, PO Box 85500, 3508 GA Utrecht, The Netherlands; 3 Netherlands Olympic Committee and Netherlands Sports Federation (NOC*NSF), PO Box 302, 6800 AH Arnhem, The Netherlands; 4 Scientific Center for Transformation in Care and Welfare (Tranzo), Tilburg University, PO Box 90153, 5000 LE Tilburg, The Netherlands

**Keywords:** Organized sports setting, Sporting program, Implementation, Health-enhancing physical activity promotion

## Abstract

**Background:**

The organized sports sector has received increased attention as a setting to promote health-enhancing physical activity (HEPA) to the general population. For significant public health impact, it is important that successful HEPA programs are widely adopted, implemented and continued as ongoing practice. The importance of evaluating the context in which programs are implemented has been identified as critical. However, little research has focused on understanding the organized sports implementation context, including factors facilitating and impeding implementation. In this study, the main factors influencing implementation of HEPA programs in the organized sports setting were studied.

**Methods:**

Fourteen sporting programs in the Netherlands aimed at increasing participation in sports by inactive population groups and funded within the National Action Plan for Sport and Exercise (NAPSE) were investigated. The programs were developed by ten Dutch National Sports Federations (NSFs) and implemented by different sports clubs in the Netherlands over a 3-year implementation period (June 2008–June 2011). The qualitative research component involved yearly face-to-face interviews (i.e. fourteen interviews each year, *n* = 12 program coordinators) and a group meeting with the program coordinators of the NSFs (*n* = 8). Cross-case comparisons and thematic analyses were performed to identify and categorize important facilitating and impeding factors respectively. The quantitative research component, used to identify the most important facilitating and impeding factors across all sporting programs, consisted of ranking of factors according to importance by the program coordinators (*n* = 12).

**Results:**

Different factors act during six identified (implementation) phases. When comparing factors across phases, several key learnings were evident. Successful implementation relied, for example, on program design and enthusiastic individuals within sporting organizations. On the other hand, inactive people were hard to reach and participation of sports clubs was not self-evident. The findings were discussed in a broader context.

**Conclusions:**

This study adds to the knowledge base concerning the implementation of sporting programs, aimed at inactive people, in the organized sports setting. The main factors facilitating and impeding implementation were identified. The results of this study can be used by sports practitioners and policy makers when developing and implementing HEPA programs in this setting.

**Electronic supplementary material:**

The online version of this article (doi:10.1186/s13102-015-0007-8) contains supplementary material, which is available to authorized users.

## Background

Participation in regular physical activity impacts positively on physical and mental health [1–3]. However, an alarming number of people are not sufficiently active to receive these benefits. In the Netherlands, this comprises 34 % of adults and 51 % of children and youth [4]. Worldwide, 31 % of adults do not engage in enough physical activity. According to the World Health Organization, insufficient physical activity is the fourth leading risk factor for mortality causing an estimated 3.2 million deaths globally each year [5]. The greatest improvements in health are obtained by increasing physical activity levels of the most inactive people, rather than getting those already active to do a little more [3].

Given the low levels of physical activity, many countries are investing resources in strategies to increase population-wide levels of health-enhancing physical activity (HEPA) [6]. Recognizing the complexity of physical activity behavior and the multiple factors influencing this behavior, more attention is paid to holistic and multi-disciplinary approaches to physical activity promotion. One such approach commonly used in health promotion is the settings-based approach, which is based on the idea that changes in people’s health and health behavior are easier to achieve if health promoters focus on settings instead of individuals [7]. This approach builds on the Ottawa Charter of 1986 that stated: “Health is created and lived by people within the settings of their everyday life; where they learn, work, play and love [8].” The settings-based approach has an ecological perspective and acknowledges the multiple levels of influence on behavior, i.e. personal, organizational, environmental and policy. It, therefore, takes into account the complexity of systems and societies in which people make their health choices [9–12]. The settings-based approach has been applied to different settings, like schools and workplaces [7, 9, 10]. A setting that has received increased attention in promoting health, including HEPA, is the organized sports sector [13–23].

There is large potential for the organized sports sector as a setting in which to promote HEPA to the general population, given the large numbers of participants, the extent of community reach and the availability of many different sports. The Dutch sports system, for example, consists of 76 National Sports Federations, approximately 25,000 sports clubs and 4.8 million sports club members [24]. Another positive aspect of participation in organized sports is that those people who are involved in organized sports are significantly more likely to meet levels of HEPA than those who are not [4]. Moreover, it has been suggested that participation in sports clubs is associated with improved psychosocial health in addition to improvements (in health) attributable to participation in physical activity [25]. Also, the social and informal nature of the sports setting has been argued to be advantageous for promoting HEPA [22, 26]. Therefore, further increasing physical activity levels of sports participants who do not meet levels of HEPA and increasing participation in sports by inactive population groups seems to be a promising strategy to enhance public health.

Different countries have already been investing resources in the organized sports sector for promoting health. Good examples of strategies can be derived from Australia. Early Australian efforts, for example, focused on delivering health promotion messages at sponsored sporting events [21]. More recently, the focus is on the creation of healthy (e.g. smoke-free settings, healthy food choices) and welcoming sporting environments as a means to increase participation in sport for health benefits [14–16, 19]. In Finland, guidelines have been developed for youth sports clubs to develop, implement and assess health promotion within their activities [23]. Another example is the “11 for Health” program, a football-based health education program, which was developed by the Fédération Internationale de Football Association (FIFA) Medical Assessment and Research Center for children in Africa. The program combined learning football skills with health education messages and was implemented in different countries in Africa [27]. Furthermore, in the Netherlands, the Dutch Ministry of Health, Welfare and Sport initiated the National Action Plan for Sport and Exercise (NAPSE). This program was aimed at increasing the number of Dutch people meeting levels of HEPA [18]. Within the NAPSE, National Sports Federations (NSFs) were asked to develop sporting programs aimed at increasing participation in sports by inactive population groups. Seventeen NSFs developed twenty-four programs which were pilot tested by a dozen local sports clubs. Based on results of a process evaluation and monitoring study [28], fourteen programs of ten NSFs were funded to be implemented more broadly by sports clubs in the Netherlands over a 3-year implementation period. Examples include a 6-week training program for adult novice runners and an adjusted form of weekly hockey for seniors played with soft balls and soft sticks.

For significant public health impact, it is important that successful HEPA programs are widely adopted, implemented and continued as ongoing practice. In this regard, the importance of evaluating the context in which programs are implemented has been identified as critical [29–33]. The primary provider of sport in the Netherlands and in many other countries (e.g. Australia, Finland, Norway) is the sports club. Any HEPA sporting program to be implemented must be interpreted and implemented by the representatives of the sports club, which are mainly volunteers and whose main focus is on providing sports activities and organizing sports competitions [32, 33]. Nonetheless, sports clubs are aware of the healthy outcomes of sport and sometimes use this as legitimacy for their activities [33].

To date, little research has focused on understanding the organized sports implementation context, including factors facilitating and impeding implementation of health programs or activities [29–31]. A few studies have been conducted in Australia concerning the development of healthy and welcoming environments (HWEs) in sports clubs [14–16, 19]. Important factors facilitating the implementation of HWEs, both for the funded State Sporting Associations and implementing sports clubs, were the availability of funding; guidance and support (e.g. training, advice, materials); understanding of benefits (i.e. increased participation at sports clubs); a positive attitude towards the HWE concept; and support of key individuals within the organization. The barriers to implementation were mainly the inverse of the facilitators. Other important barriers were State Sporting Associations’ limited capacity and power to influence activities at the club level; limited capacity of volunteer-based sports clubs; no clear outlining of expectations and responsibilities; unrealistic time-frames for implementation; and structural impediments (e.g. a lack of facilities, costs). Furthermore, the Finnish “Health Promoting Sports Club” guidelines provide fourteen consecutive steps for local youth sports clubs to enhance health promotion as part of their activities [23]. The guidelines are divided into policy development actions and activities a club needs to perform before the club actors (e.g. coaches) can implement health promotion as part of their daily practice, like “prioritize the most relevant health promotion aims” (policy development) and “educate coaches and other club officials” (practice development). For each guideline, the rationale and practical examples are provided.

The aforementioned studies have a relatively broad focus when it comes to health promotion. Less is known about the implementation of HEPA programs per se. The promotion of HEPA is more closely related to the core business of sporting organizations (i.e. the provision of sports activities) than other health promotion actions and may, therefore, result in different implementation successes or challenges. There is one Australian study that focused on partnership and capacity-building strategies associated with successful implementation of cross-sectoral (e.g. sports, recreation, health) sports and recreation programs [13]. The investigated programs had a strong emphasis on participation in physical activity that would benefit people who were not currently active and on low incomes. The findings showed that engagement of key stakeholders, formalization of the partnership agreement and capacity (diversity of skills and resources within the partnership) to develop and implement sports and recreation programs facilitated program implementation. In addition, addressing the development of partnerships, implementing a phased approach to program development and implementation was suggested to assist the sports and recreation sector build capacity to participate in partnership approaches to health promotion. The researchers focused on cross-sectoral HEPA programs and addressed only a particular aspect of the implementation process (i.e. partnership and capacity-building strategies). Therefore, to gain a broader understanding of the implementation of HEPA programs in the organized sports setting, this study focused on HEPA programs initiated by sporting organizations and the implementation process as a whole. Specifically, the main factors influencing implementation of the fourteen NAPSE sporting programs, initiated by NSFs and implemented by sports clubs in the Netherlands, were studied. The study results will support sports practitioners and policy makers with developing and implementing HEPA programs in this setting. The findings of this study are of international interest, particularly in countries where the organized sports sector is used as a setting to promote HEPA.

## Methods

### Sample

This study focused on the fourteen NAPSE sporting programs and the ten funded NSFs, i.e. the program coordinators who were designated to facilitate implementation of these programs in local sports clubs in the Netherlands. The programs varied with regard to targeted age group, content and duration. A description of the programs can be found in Table 1. The NAPSE sporting programs were implemented by different sports clubs over a 3-year implementation period (June 2008–June 2011). Sizes of the NSFs, as well as program aims and the extent to which NSFs were successful in achieving these aims, varied widely. The actual reach of the programs ranged from 9 (45 % of aim reached) to 680 (159 %) participating locations (a sports club could implement the program in different locations) and 85 (43 %) to 273,896 (304 %) participants (see Table 2).

**Table 1:** Description Dutch NAPSE sporting programs

NSF	Sporting program	Target group	Description
Athletics	Start to Run	Adults	Six-week training program for novice runners aimed at running 3 km continuously. The program is offered by athletics clubs and running stores.
Judo	Judo in school	Children, adolescents	During a few weeks judo lessons in school provided by a qualified judo trainer.
Walking	Through 4 days Marches	Adults	Six-month training program for the Four Days Marches of Nijmegen. Participants can take part in the program individually or at a walking club.
Walking	Working by Walking	Adults	Walking program of at least 16 weeks aimed at improving health parameters. The program is provided by qualified walking trainers.
Gymnastics	Trendy Weeks for Masters	Older adults (45+)	During 8–12 weeks gymnastic classes with a specific theme (e.g. Move on music) at a gymnastics club.
Hockey	Fit Hockey	Older adults (50+)	Hockey played in a team with soft sticks and soft balls; training opportunities are provided continuously at the hockey club.
Swimming	My Swimming Coach	Adults	A membership of the NSF, including access to an online swimming coach and opportunities to participate in swim clinics and events.
Bridge	Thinking and Doing	Older adults (55+)	A project of two years in which bridge is used to create communities of older people. After a year physical activities are offered.
Sportive cycling	Cycle-Fit	Adults	Six-week training program for novice cyclers (speed cycling, mountain biking). The program is offered by (sportive) cycling clubs and cycling stores.
Sportive cycling	Cycle & Enjoy Nature	Older adults (45+)	Regular recreational cycling activities with a focus on relaxing and enjoying nature at a cycling club; or and individual introduction package including a cycling magazine, a training manual, a map with cycling routes and a calendar with cycling events.
Triathlon	Trio-Triathlon	Adults	Organization of Trio-Triathlon (the three sports of a triathlon are performed by three different individuals) events.
Volleyball	Beach volleyball	Children, adolescents, adults	Organization of different beach volleyball activities (e.g. clinics, tournaments, workshops) at schools, (beach) volleyball clubs, companies and (beach) volleyball events.
Volleyball	Cool Moves Volley	Children	A volleyball approach adapted to the abilities and needs of kids. Training opportunities are provided continuously at volleyball clubs; clinics are provided in schools.
Volleyball	Ultimate Volley Xperience	Adolescents	A volleyball event in a Caribbean atmosphere. The event is held at a special location and includes music and spectacular side-events.

**Table 2:** Size NSFs and reach programs in relation to aims

NSF	Size NSF^a^	Sporting program	Locations*: aim (N)^b^	Locations*: result (N, % of aim)	Participants: aim (N)^c^	Participants: result (N, % of aim)
Athletics	Large	Start to Run	150	120 (80 %)	26.000	25.777 (99 %)
Judo	Medium	Judo in school	No aim	474 (NA)	10.000	53.804 (538 %)
Walking	Medium	Through 4 days Marches	NA	NA	3.500	4.650 (133 %)
Walking	Medium	Working by Walking	20	9 (45 %)	200	85 (43 %)
Gymnastics	Large	Trendy Weeks for Masters	220	218 (99 %)	7.500	3.110 (41 %)
Hockey	Large	Fit Hockey	20	15 (75 %)	1.000	989 (99 %)
Swimming	Large	My Swimming Coach	130	70 (54 %)	19.000	11.350 (60 %)
Bridge	Large	Thinking and Doing	38	71 (187 %)	2.925	4.055 (139 %)
Sportive cycling	Medium	Cycle-Fit	420	228 (54 %)	8.000	3.057 (38 %)
Sportive cycling	Medium	Cycle & Enjoy Nature	No aim	47 (NA)	1.000	446 (45 %)
Triathlon	Small	Trio-Triathlon	40	66 (165 %)	9.600	13.014 (136 %)
Volleyball	Large	Beach volleyball	52	104 (200 %)	90.000	273.896 (304 %)
Volleyball	Large	Cool Moves Volley	428	680 (159 %)	14.000	49.883 (356 %)
Volleyball	Large	Ultimate Volley Xperience	220	164 (75 %)	56.750	34.658 (61 %)

^*^A sports club could implement the program in different locations

^a^Large > 100,000 club members, medium 25,000–100,000 club members, small < 25,000 club members

^b^Aimed number of locations in which the sporting program will be implemented

^c^Aimed number of participants of sporting program

NA = not applicable

### Design

This research was part of a larger study in which both a process and effectiveness evaluation of the programs were conducted [34]. It consisted of a qualitative component to explore factors facilitating and impeding implementation of the individual sporting programs followed by a quantitative component to identify a generic set of factors (i.e. the most important factors across all sporting programs) influencing implementation. The qualitative component results informed the quantitative component. When performing the study, ethical guidelines were followed (i.e. with regard to avoiding undue intrusion, avoiding adverse consequences, confidentiality, enabling participation, informed consent and data protection) [35]. Before the start of the implementation phase, a group meeting was held with the program coordinators of the NSFs to explain in plain language the purpose of the research, the methods, demands, potential risks and possible outcomes of the research. In addition, it was explained that monitoring implementation progress was part of the NAPSE funding agreement, but that NSFs were not judged on the basis of the program or research results (i.e. the NSFs had a best-efforts obligation with regard to implementing the programs). Written informed consent for participation in the study was obtained from the NSFs when they applied for the funding. According to Dutch legislation, approval by a medical ethics committee was not obligatory, as participants were not subjected to procedures, nor were they required to follow rules of behavior. The privacy regulations of the study were approved by the Dutch Data Protection Authority. For reporting of results, the RATS guidelines were used as a guidance [36].

## Procedures

### Qualitative part

#### Face-to-face interviews

Face-to-face interviews were conducted with the program coordinators of the NSFs (*n* = 12; one program coordinator per NAPSE sporting program; two program coordinators were responsible for two programs). Overall, fourteen (i.e. one interview per program) semi-structured interviews of 60–90 min duration were conducted yearly (in 2009, 2010 and 2011) during the 3-year implementation period by the primary researcher (LO). The same program coordinators participated in the interviews, except for five sporting programs. For these programs, there was a change in program coordinator during the implementation period. The interview questions focused on implementation progress in the past year and factors that respondents believed have facilitated and/or hindered implementation. Also, other topics were considered, like the number of participants, obtained results, developed products, collaboration with other parties, the follow-up activities offered and continuation of the program after cessation of funding. The latter topics, however, were asked in light of the general process evaluation. In this research, they were only used as background information. The interview questions were partly based on interview questions used during the pilot study [28] and further developed by an expert panel consisting of the two researchers (LO and CV) and representatives of the Netherlands Olympic Committee and Netherlands Sports Federation (NOC*NSF), the Dutch Ministry of Health, Welfare and Sport, the Netherlands Institute for Sport and Physical Activity and four NSFs. The interviews were recorded with a digital voice recorder and transcribed later on. A transcript summarizing the main findings was sent for review and revision to the interviewees.

#### Extraction and categorizing of factors

For each sporting program, facilitating and impeding factors were extracted from the interview transcripts and summarized. Subsequently, a list of facilitating and impeding factors for all sporting programs was created. For this purpose, cross case analyses [37] were performed with the individual sporting programs representing the cases. Factors were compared between sporting programs to investigate commonalities: Comparable factors between sporting programs were summarized and added as a single factor. For instance, multiple program coordinators indicated that it was important to visit sports clubs personally when asking for participation. This was summarized as the facilitating factor: “approaching sports clubs personally”. Unique factors were added as a single factor to the list. Finally, thematic analyses [38] were performed to categorize the factors according to themes. Extraction of factors, cross-case and thematic analyses were performed manually by the primary researcher (LO) using Microsoft Word 2010 (Microsoft Corporation, Redmond, United States). To enhance rigor, all analyses were checked by a second researcher (CV). Differences between researchers were discussed. This resulted in only minor adjustments.

#### Meeting with program coordinators

To verify the obtained factors and categorizing according to themes or, in this case, phases, the results were presented and discussed at a meeting with the program coordinators (*n* = 8, the others were unable to attend). In a general discussion which was led by the primary researcher (LO), consensus was reached about the phases. In addition, the program coordinators were asked whether they agreed on the identified factors and whether any important factors were missing. For this purpose, they circulated along paper boards. On each board, a phase with its accompanying factors was presented. Program coordinators could add or remove factors. This led to the addition of 16 new facilitating factors and three new impeding factors. These factors are presented in bold in Additional file 1 [See Additional file 1 - Overview of all factors and their ranking scores]. No factors were removed. Subsequently, a final overview of factors categorized by phases was made by the researcher (LO).

### Quantitative part

#### Ranking of factors

To identify the most important facilitating and impeding factors across all sporting programs, the final overview was sent to the program coordinators (*n* = 12) by email. The program coordinators were asked to rank the factors in an attached ranking form, whereby ranking was done by phase and for facilitating and impeding factors separately. The most important (facilitating or impeding) factor was assigned ranking 1, the second most important factor was assigned ranking 2, etc. As the number of factors varied by phase, the number of assigned rankings also varied. Completed ranking forms (*n* = 12) were returned by email.

#### Calculating mean ranking scores and composing the top three of factors

The mean ranking score for each factor was calculated (i.e. sum of ranks divided by number of program coordinators (*n* = 12)) by the researcher (LO) using Stata statistical software version 10.1 (Stata Corporation, College Station, Texas). Then, the factors were placed in order of importance, with the lower mean ranking scores corresponding to more important factors. Subsequently, the top three facilitating and impeding factors were composed per phase. In case there were three or less than three factors, all factors are presented.

## Results

### Phases and final overview of factors

Based on thematic analyses and the meeting with the program coordinators of the NSFs, factors were categorized according to six phases through which all programs proceeded in a consecutive manner:

1. Program development: factors that influenced implementation, but had to be dealt with during program development, i.e. the phase proceeding implementation;
2. Organizational (pre)conditions: factors that influenced implementation at the level of the NSF;
3. Recruiting local sports clubs: factors that affected the recruitment of local sports clubs;
4. Recruiting participants: factors that facilitated or impeded recruitment of participants for the program;
5. Local implementation: factors that were important during local implementation, i.e. at the level of the sports club;
6. Securing continuation of the program: factors that had to be considered during implementation and influenced continuation of the program after implementation, both at the level of the NSF and sports clubs.

The final overview of factors, comprising the six phases, contained a total of 56 facilitating and 29 impeding factors [See Additional file 1 - Overview of all factors and their ranking scores]. The number of facilitating factors varied by phase from 7 to 12, the number of impeding factors varied by implementation phase from 2 to 7. In each phase, there were more facilitating than impeding factors.

### Ranking results

Based on the ranking of factors according to importance, the top three facilitating and impeding factors per phase were identified. These results are presented in Tables 3 and 4 for facilitating and impeding factors respectively. Comparing both tables, it is apparent that the impeding factors were mainly the inverse of the facilitating factors. Furthermore, the range of rankings shows that there were some differences in ranking by the program coordinators. Under the headings of the six phases, the factors will be explained in more detail and illustrated with examples provided by the program coordinators in the interviews. Since the impeding factors were often the inverse of the facilitators, they are not always explained separately. In the additional file, all factors and their ranking scores are presented [See Additional file 1 - Overview of all factors and their ranking scores].

**Table 3:** The top three facilitating factors per phase based on ranking by NAPSE program coordinators (*n* = 12)

Phase (total number of factors in phase)	Top three factors	Mean ranking score^a^	Range assigned rankings^b^
1. Program development (*n* = 12)	• The program matches the target group’s needs, wishes and possibilities	1.1	1–2
	• The program is easy to implement locally	3.8	2–7
	• Low threshold for participation of inactive people	3.9	1–7
2. Organizational (pre)conditions (*n* = 10)	• Having a “dedicated” program coordinator	2.9	1–8
	• Sufficient time (in man-hours) to coordinate the program	3.1	1–6
	• Internal support for the program	3.9	1–9
3. Recruiting local sports clubs (*n* = 10)	• Providing a complete (readily usable) package to sports clubs	3.8	1–7
	• Approaching sports clubs personally	4.3	1–10
	• Support for the program by sports clubs	4.7	1–10
4. Recruiting participants (*n* = 9)	• Support for the program by the target group	3.3	1–6
	• A good promotion/marketing strategy nationally and locally	3.4	1–8
	• The sports activities are organized in close proximity to the target group	3.6	1–6
5. Local implementation (*n* = 8)	• Enthusiastic people within sports clubs delivering (high-)quality performances	2.9	1–7
	• Sports clubs are (personally) supported by the NSF when implementing the program locally	3.8	1–7
	• Availability of follow-up sports activities locally that match participants’ needs, wishes and possibilities	3.8	1–8
6. Securing continuation of the program (*n* = 7)	• The program is part of the NSF’s long-term policy	2.6	1–5
	• The NSF has sufficient financial resources available to continue the program/secure the program for the future	2.7	1–6
	• The program is part of the sports club’s long-term policy	3.0	1–6

^a^For each factor: Sum of rankings divided by the number of program coordinators (*n* = 12)

^b^Lowest and highest ranking of factor

**Table 4:** The top three impeding factors per phase based on ranking by NAPSE program coordinators (*n* = 12)

Phase (total number of factors in phase)	Top three factors	Mean ranking score^a^	Range assigned rankings^b^
1. Program development (*n* = 6)	• The program does not match the target group’s needs, wishes and/or possibilities	1.8	1–6
	• The (implementation of the) program (locally) is costly	3.2	1–5
	• The program does not match the needs, wishes and/or possibilities of sports clubs	3.3	1–5
2. Organizational (pre)conditions (*n* = 3)	• Insufficient finances to coordinate and implement the program	1.4	1-2
	• No or insufficient support for the program internally	1.9	1–3
	• Internal organizational changes	2.7	1–3
3. Recruiting local sports clubs (*n* = 6)	• No or insufficient qualified trainers locally	2.2	1–4
	• No or insufficient support for the program by sports clubs	2.4	1–6
	• Unavailability of additional (local) funding possibilities	3.6	1–6
4. Recruiting participants (*n* = 7)	• The target group is unfamiliar with the program or the sport	3.0	1–5
	• No or insufficient support for the program by the target group	3.3	1–7
	• The program does not reach/engage inactive people	3.4	1–5
5. Local implementation (*n* = 5)	• No enthusiastic and/or incompetent people within sports clubs	1.5	1–4
	• No clear division of roles, tasks and responsibilities between the NSF and sports clubs	3.0	1–5
	• No (appropriate) follow-up sports activities for participants locally	3.2	1–5
6. Securing continuation of the program (*n* = 2)	• The NSF has insufficient financial resources available to continue the program/secure the program for the future	1.4	1–2
	• Sports clubs have insufficient financial resources available to continue the program locally/secure the program for the future	1.6	1–2

^a^For each factor: Sum of rankings divided by the number of program coordinators (*n* = 12)

^b^Lowest and highest ranking of factor

### Program development

It was reported that, when developing a program, it is important to consider the needs, wishes and possibilities of the target group. Both the content of the program (e.g. sport, intensity of activity) as well as organizational aspects (e.g. day and time of activities) have to be tailored to the target group.

The NAPSE sporting programs were aimed at inactive people. For this particular target group, the threshold for participation had to be low. This meant that people with no previous training experiences or specific sport skills could participate and sports activities were offered in a non-threatening manner to non-sport participants. The NSFs lowered barriers for participation by using graded training programs (i.e. starting with small amounts of physical activity and gradually increasing intensity over time), simplified sport techniques and/or rules and easy to use (soft and non-threatening) sport materials.

Furthermore, it was important to consider the needs, wishes and possibilities of local sports clubs, because they were the ones actually implementing the programs. The main focus of sports clubs was to provide sports competition and they generally relied on volunteers. Implementation was facilitated when the program was easy to implement locally, i.e. the program required little in terms of materials, manpower and time. In contrast, high implementation costs (i.e. a costly program) were perceived as a barrier to local implementation. For example, intensive personal guidance of participants, expensive program materials, (regular) transportation of sports equipment and the need to rent a specific (sports) accommodation contributed to high implementation costs.

### Organizational (pre)conditions

A major facilitator of implementation at the level of the NSF was having a “dedicated” program coordinator, i.e. someone who was committed to the program and believed in it. Additionally, it was important that this person had sufficient time to coordinate the program. Preferably, he or she was only working on the program and was not being distracted by other projects or activities.

Another key factor to successful implementation at the level of the NSF was having support for the program within the NSF’s organization of both individuals that were involved in the program as well as individuals that were not directly involved (e.g. staff members and other NSF departments). High level management and administrative commitment and support was especially needed in cases where the program required large organizational or structural changes. For example, for one sporting program a new type of membership was introduced, whereby individual sports participants became a direct member of the NSF. In absence of support, it took longer to get programs running and, in some cases, this led to delayed implementation.

Other main factors at the level of the NSF impeding implementation were insufficient finances for coordination/implementation and internal organizational changes. The NAPSE programs were all funded programs. For the implementation of two programs additional co-financing of municipalities was taken into account, so that the programs could be implemented in more locations than was possible with the NAPSE funding alone. Many municipalities have a budget to stimulate sports participation. However, municipalities were not always willing or able (i.e. there was no budget or the budget was already spent) to provide financial resources, which hindered the implementation of the programs in certain locations. Next to that, the NSFs agreed that, in general, insufficient finances are an important barrier to implementation of these kind of sporting programs. Examples of (unfavorable) organizational changes that took place were staff turnover, policy changes and a reorganization of the NSF. Particularly, a change of program coordinator resulted in slowed implementation of planned activities within the programs. This was due to the time required for the NSF to employ a new program coordinator and for the new program coordinator to become oriented with the program and sports clubs.

### Recruiting local sports clubs

Local sports clubs were more willing to participate when they valued the program and supported it. The opposite, was a great barrier to recruiting sports clubs. Most NSFs experienced (some) resistance from their sports clubs, especially when the programs were first introduced. The NSFs described the sports clubs as being traditional: Their primary focus was on running their sports competitions and regular training programs. Providing physical activity opportunities for inactive people was something else and beyond their core business. However, during the 3-year implementation period, resistance often ceased because sports clubs became familiar with the program and/or were positively affected by other sports clubs implementing the program. Sports clubs were introduced to the successes of other sports clubs through the NSF website, the NSF (online) newsletter, adverts in local newspapers and (meetings with) trainers of other sports clubs. Also, different sports clubs provided demonstrations of the sporting programs (e.g. during large sports events) and implementation successes were spread by partner organizations of the NSF and implementing sports clubs.

Nonetheless, to convince sports clubs to participate a more personal approach was required. Preferably, the NSF visited the club personally to explain the program and ask for participation. Furthermore, a complete (readily usable) package with all necessary materials (e.g. trainer manual, sport materials, promotional materials) enhanced participation of sports clubs, because this saved them a lot of time during local implementation.

A main factor impeding participation of sports clubs was having no or insufficient qualified trainers. Finding volunteers with the appropriate skills (i.e. volunteers that were able to work with non-sport participants) was an issue for many sports clubs. Most NSFs developed and organized special trainer courses to grow their trainer database.

For implementation of the programs, it was sometimes required that sports clubs invested their own financial resources (e.g. to produce promotional materials, pay trainers or buy sports equipment). However, sports clubs did not always have enough internal financial resources and they depended on external financial resources to participate. Unavailability of (local) funding possibilities, therefore, impeded the recruitment of sports clubs as well.

### Recruiting participants

As with the recruitment of sports clubs, recruitment of participants was facilitated when (potential) participants valued the program and supported it. In addition, a good promotion and marketing strategy, both nationally and locally, was needed. According to the NSFs, promoting the program at a national level was necessary to get people familiar with the program. For the actual recruitment of participants, however, local marketing was more important. The NSFs marketed their programs via national press, the internet, television and partner organizations. Local marketing was done by sports clubs through the distribution of posters, flyers and leaflets. Also, adverts were placed in local newspapers, demonstrations were given and participants were recruited by word of mouth. On the other hand, when the target group was unfamiliar with the program or the sport in general, recruitment was impeded. Some sports, for example, had the image of being tough (e.g. hockey, sports of a triathlon) or only to be played by certain (sub)groups (e.g. hockey).

Organizing the sports activities in close proximity to the target group was another factor facilitating recruitment. In this way, travel distance was no barrier to participation. Providing the program in many different locations in the Netherlands was one way that some NSFs dealt with this. Others organized the sports activities in places where the target group gathered; for example, activities for children were organized in elementary schools.

Finally, the NAPSE sporting programs were all aimed at increasing physical activity levels of inactive people. However, some NSFs stated that it was very difficult to attract large numbers of inactive people to their sports activities. Most participants were already a little or very physically active. A NSF summarized this as not having the right people and channels to reach this target group.

### Local implementation

For the program to be successfully implemented by a local sports club, it was important to have people (e.g. a trainer) within the club who were enthusiastic about the program and skilled in running the program. The NSFs stated that, when the program was of high-quality (i.e. participants had good experiences due to trainer capabilities), participants gladly came back to the club to participate in (additional) sports activities.

(Personal) support of sports clubs by the NSF was another factor positively influencing local implementation. During the implementation period, the NSFs supported sports clubs in different ways, for example, by providing them with personal guidance, financial resources, advice and (promotional) materials. In this way, local barriers were overcome and sports clubs spent less time on decision making and developing new products.

A main factor hindering implementation locally was an unclear division of roles, tasks and responsibilities between the NSF and sports clubs. For the NSF, this resulted in doing more supportive work than initially planned; and for the sport club, this led to a slowing down of planned activities.

Finally, to stimulate continuous participation in physical activity and to recruit new members for the sports club, it was important to have follow-up activities at the sports club that matched participants’ needs, wishes and possibilities. Follow-up activities consisted, for example, of a few additional introductory training sessions for free or at low costs at the club. Sports clubs also offered membership options at a reduced rate with continuation of activities in an appropriate beginners’ group. In cases where there were no suitable follow-up activities at the sports club, it was difficult to capture participants’ interest for the sport and they were often lost to club membership.

### Securing continuation of the program

Including the program in both the NSF’s and sports club’s long-term policy was reported to enhance continuation of the program. This ensured integration of the program in the organization and available time and resources to run the program. With regard to resources, having sufficient financial resources was reported as a separate facilitating factor. At the same time, a lack of financial resources was seen as a great barrier to sustaining a program, both for the NSF as well as the sports clubs. Of the fourteen NAPSE sporting programs, ten were reported to be continued after the funding period. Programs that were self-sustaining after the implementation period spent funds to develop the program infrastructure, such as educating trainers and providing equipment and resources. These programs could be financed from internal financial resources and/or membership/participation fees. Other programs still relied on external financial resources following cessation of funding. In these cases, the program infrastructure was not fully developed and program costs (e.g. costs related to use of accommodations or facilities, sports equipment, payment of trainers, promotion and recruitment strategies) could not be covered by internal financial resources or membership/participation fees. At the time of the last interview, some NSFs were still struggling with finding sponsors or funding opportunities. Others already found a sponsor for their programs for the next year, like a health insurance company and an international bank.

### Summary of results

**Fig. 1:**
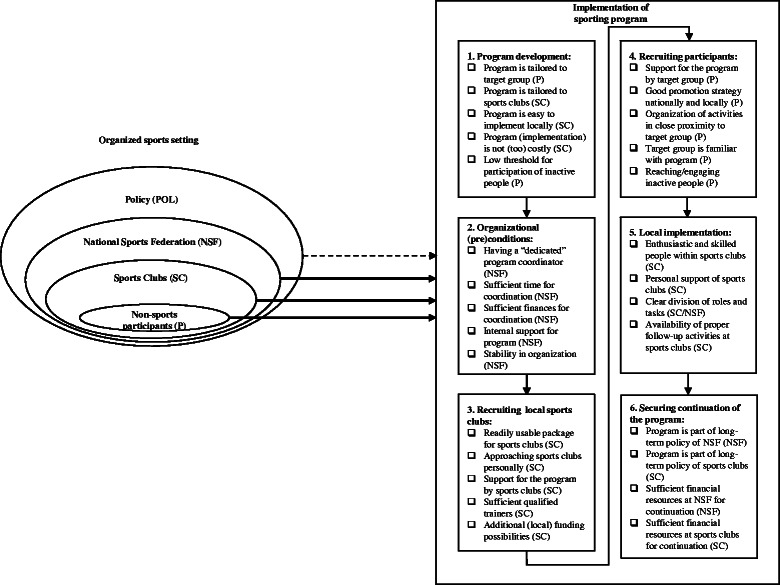
Summary of results in an ecological model

Given the ecological perspective of the settings-based approach [9–12], an ecological model is used to summarize the results (see Fig. 1). The organized sports setting and the different levels of influence - policy, NSF and sports clubs (organizational and environmental level) and non-sport participants (personal level) - are presented on the left. The factors are presented on the right in the form of a checklist, which can be used as guidance when developing and implementing HEPA programs in the organized sports setting. The arrows indicate the influence of the different ecological levels on the implementation process. Behind each factor, the exact level of influence is indicated (NSF, SC, P). For instance, at the non-sport participants and sports club level, program development must consider how the program can be tailored to the target group (inactive people) and sports clubs; and at the non-sport participants level, recruitment must consider both national and local promotion strategies. Furthermore, the results show that the major factors influencing implementation of HEPA programs by the organized sports setting do not act on the policy level (e.g. regulations for facilities, safety laws). Therefore, the arrow representing this influence is dashed.

## Discussion

A new setting of interest in the promotion of health, including health-enhancing physical activity (HEPA), is the organized sports sector [13–23]. In this study, the main factors facilitating and impeding implementation of sporting programs, aimed at inactive population groups, were identified (see Tables 3, 4 and Fig. 1). The results showed that different factors acted during the different phases of the implementation process. When comparing factors across phases, several key learnings are evident. These will be discussed here in a broader context.

First, program design is an important influencer of implementation. HEPA sporting programs must be tailored to the needs, wishes and possibilities of both inactive populations groups as well as the implementing sports clubs. This could be achieved by engaging and actively involving these parties in the program development and implementation process [39]. Consequently, support for and implementation of the program will be enhanced. In addition, there are different formative research strategies (e.g. interviews, observations, focus groups) that can be used before a program is developed or implemented to obtain detailed information about the target group and implementers of the program [40]. In this study, the focus was on implementation and not on program development. Therefore, it is not known how the programs were developed and whether non-sport participants and sports clubs were involved in program design. However, all programs were pilot tested before advancing to broader implementation [28], which suggests that needs of both participants and sports clubs have been addressed. Satisfaction of participants with the programs was measured during the effectiveness evaluation of the programs [34]. The results showed that a majority (87–99 %) of participants of the sporting programs enjoyed participation and liked the sports activities offered. The average rating of the programs varied between 7.6 and 8.4 (scale 0–10; 0 being very poor and 10 being excellent), which indicates that the sporting programs were suitable for their participants.

However, the actual target group, i.e. inactive people, was not always reached, at least not in great numbers. It seems that the organized sports sector is not yet “a setting of everyday life” for this target group. The results of the effectiveness evaluation confirm this [34]. The percentage of inactive people varied from 0 to 15 % per sporting program. In addition, for seven sporting programs enough data were available to assess effectiveness on increasing levels of HEPA. Three sporting programs showed significant increases in physical activity levels of participants six months after the start of the programs and in comparison with a control group. For two of these programs, this was accompanied by a significant increase in the percentage of participants meeting levels of HEPA (+26 and +12 % compared to baseline). For the remaining four sporting programs, no significant changes in levels of physical activity of participants were observed. Therefore, to engage more inactive people into organized sports activities, sporting organizations should focus on proper recruitment methods and channels to reach this target group. In this regard, they may consider engaging in partnerships with primary health care, community health or other relevant organizations to get closer to this target group [41].

Another important finding was that successful implementation was largely dependent upon enthusiastic people within sporting organizations (e.g. program coordinator, trainer at a sports club) that were willing to invest time in coordinating/running the program. These people needed to possess the right skills, especially the local trainers, to provide high-quality training programs. This ensured participants returned to the club for (additional) sports activities.

For continued participation in sport of (initially) inactive people, however, suitable follow-up activities were required at the sports club. This was also advocated in a study which investigated structural links between sports participation programs conducted in schools and participation in community-based sports clubs [42]. The school-based sporting programs were seen as ineffective in promoting sustained sports participation and club membership due to a lack of formal strategies linking program participants with sports clubs. Repeated or additional experiences in the sport at the sports club was one of the strategies suggested to encourage engagement of participants in a local sports club. In addition, it was recommended that sporting organizations tailor their school-based programs using recognized health promotion planning principles (including taking into account the needs of participants and sports clubs) rather than continuing their current “one-size-fits-all” approach.

Furthermore, within the NAPSE, the NSFs received the funding, but the sports clubs were the ones actually implementing the HEPA programs. (Financial) resources of the mainly voluntary-based sports clubs were often limited. In addition, their primary focus was on running their sports competitions and regular training programs. Therefore, it was not self-evident that sports clubs implemented the HEPA programs in addition to their regular sports activities. Similar conclusions were drawn in a study which investigated the work of sports clubs as seen by representatives of sports clubs [33]. Sports clubs do what is familiar to them and respond to their local environment [32]. In the current study, it was also seen that sports clubs’ support for the programs increased when they became familiar with the programs and were introduced to successes of other sports clubs implementing the programs. These latter sports clubs can be seen as the “early adopters” in the diffusion of innovations theory [43] and can thus be used to recruit other sports clubs. Nonetheless, to convince sports clubs to actually participate a more personal approach was required. Moreover, the support that NSFs offered to sports clubs (i.e. providing sports clubs with personal guidance, financial resources, advice and (promotional) materials) was essential and facilitated implementation at the sports club level. At the same time, this highlights the need to allocate (financial) resources directly to the implementing sports club and it should be considered in funding arrangements in which the implementing sports club is not the receiver of the funding.

Finally, for population health gains, all programs for promoting HEPA through sport will need to be sustained over a long period of time [17]. Of the fourteen NAPSE programs, ten were reported to be continued after the funding period. A lack of financial resources was seen as a great barrier to sustaining a program, both for the NSFs as well as the sports clubs. The sustainability of health promotion programs within sport and recreation organizations also relied heavily on continued funding [44]. Hence, it is important that program funds are spent to develop the program infrastructure so that the program is self-sustaining and can be financed from internal financial resources and/or membership/participation fees. It should be noted that it is unknown whether the NAPSE programs will actually be sustained in the absence of ongoing funding. This was also a limitation of the study concerning the sustainability of health promotion programs within sport and recreation organizations [44]. Thus, further research is required to examine factors influencing the long-term sustainability of HEPA programs in the organized sports setting.

The findings of this study add to those found in the study regarding partnership and capacity-building strategies associated with successful implementation of cross-sectoral sport and recreation programs [13]. In addition, the identified factors are comparable to those found in the studies concerning the implementation of HWEs [14–16, 19] and the factors acting on the sports club level support the “Health Promoting Sports Club” guidelines [23]. For instance, the guideline “determine the current state of will to practice health promotion in your club” is a prerequisite for the identified facilitating factor “support for the program by sports clubs” and the guideline “educate coaches and other club officials” is the solution to the impeding factor “no or insufficient qualified trainers locally”. This implies there are generic factors, independent of the health program or activity being implemented, influencing implementation in the organized sports setting. Nonetheless, some factors are specific to the implementation of HEPA programs, such as the factor “low threshold for participation of inactive people (program development)”.

In contrast to the organized sports setting, a lot of research is available concerning the implementation of promotion and prevention programs in other settings, like schools and the health care setting (e.g. [45, 46]). Surprisingly, the identified factors in this study reflect some of the major facilitators (and barriers) to program implementation in these settings, such as the availability of funding, skill proficiency (of providers) (vs. trainers with the appropriate skills), compatibility of the innovation (vs. a program that matches the needs, wishes and possibilities of sports clubs), the existence of a program champion (vs. enthusiastic people within sporting organizations) and provision of training and technical assistance (vs. providing (personal) support to sports clubs) [45]. Nevertheless, some caution in interpreting this findings is needed. The practical realization of factors may, for example, be quite different between programs or settings (e.g. training volunteers in sports clubs to provide HEPA programs vs. training health professionals in providing substance abuse prevention programs). Therefore, the context in which programs are implemented remains important [29–31].

Overall, the findings of this study can assist sports practitioners and policy makers with developing and implementing HEPA programs in the organized sports setting. Figure 1, in which the results are summarized, can be used as guidance to tailor programs and implementation strategies to this setting. Also, the practical examples provided in the results section may be of value for realization of factors. Moreover, the results can be used to guide funding guidelines (e.g. allocating (a part of the) financial resources directly to implementing sports clubs and using funds for developing program infrastructure). Considering the main facilitating and impeding factors during the development and implementation process will concurrently facilitate successful implementation [29–31].

This study was designed to contribute to the understanding of the implementation of HEPA programs in the organized sports setting. A strength of the study was that the implementation process was studied longitudinally. Interviews were conducted yearly during the 3-year implementation period. In this way, the (time-)specific features of the implementation process were better captured and recall bias was reduced. Moreover, the (partly) qualitative nature of the study (i.e. semi-structured interviews, meeting) revealed detailed information about the implementation process. A disadvantage of self-report might be the introduction of social desirability biases. However, given the fact that the program coordinators of the NSFs reported both the facilitators and barriers to implementation, it appears there was limited social desirability bias. In addition, it is believed that the different evaluation rounds ensured the reliability of this study.

With regard to quantitative ranking of factors, the mean ranking score was calculated from an ordinal ranking scale. It is not certain whether the difference in ranking between one and two is the same as that, for example, between three and four on a ordinal ranking scale. Moreover, when the mean ranking score of factor A is two and four of factor B, this does not necessarily mean that factor A was twice as important as factor B. In addition, there were some differences in ranking of factors between program coordinators. This could be due to differences in programs (e.g. content, size) and/or NSFs (e.g. size, organizational structure), making some factors more or less relevant. However, the sum of ranks and the mode of ranks (results not presented in this article) did not yield a different ordering of factors. Therefore, it is believed that the mean ranking score was appropriate to identify the top three facilitating and impeding factors in this study.

Furthermore, in this study, the focus was on the perceptions of the program coordinators of the NSFs, because they were the ones designated to facilitate implementation of the programs in local sports clubs. Information was not directly obtained from representatives of the sports clubs, i.e. the actual implementers of the programs. This could be seen as a limitation of the study. However, the program coordinators worked in close collaboration with the sports clubs and were, therefore, well-informed about the implementation process locally. Nonetheless, in future research it would be interesting to evaluate directly at the sports club level.

## Conclusions

Considering both the strengths and limitations, this study does add to knowledge base concerning the implementation of sporting programs, aimed at inactive people, in the organized sports setting. The main factors facilitating and impeding implementation were identified. The results of this study can be used by sports practitioners and policy makers when developing and implementing HEPA programs in this setting. Moreover, the results can be used to guide funding guidelines. In future research, it would be interesting to evaluate implementation directly at the sports club level and to study factors influencing the long-term sustainability of HEPA programs. This will further contribute to the understanding of the implementation context of the organized sports setting, and will, consequently, improve the implementation and sustainability of HEPA programs in this setting.

## Additional file

Additional file 1: **Overview of all factors and their ranking scores.** In the additional file, an overview of all facilitating and impeding factors per (implementation) phase can be found, including their ranking scores (i.e. mean ranking score and range assigned rankings).

## Abbreviations

HEPA: Health-enhancing physical activity
HWEs: Healthy and welcoming sporting environments
FIFA: Fédération Internationale de Football Association
NAPSE: National Action Plan for Sport and Exercise
NOC*NSF: Netherlands Olympic Committee and Netherlands Sports Federation
NSF: National Sports Federation
P: Non-sport participants
POL: Policy
SC: Sports Clubs
