# Supplementary material for: Sporting programs for inactive population groups: factors influencing implementation in the organized sports setting
Source: BMC Sports Sci Med Rehabil. 2015 Jun 3;7:12. doi: 10.1186/s13102-015-0007-8 (PMC4451741; doi:10.1186/s13102-015-0007-8)
Supplement: Additional file 1: — Overview of all factors and their ranking scores. In the additional file, an overview of all facilitating and impeding factors per (implementation) phase can be found, including their ranking scores (i.e. mean ranking score and range assigned rankings). [file 13102_2015_7_MOESM1_ESM.docx]

**Overview of all facilitating and impeding factors per (implementation) phase, including ranking scores^[[1]](#footnote-1)^**

The factors presented in **bold** were added during the meeting with the NSF program coordinators.

Table 1: Phase 1 Program development: facilitating factors (n=12)

| **Facilitating factor** | **Mean ranking score** | **Range assigned rankings** |
| --- | --- | --- |
| - The program matches the target group’s needs, wishes and possibilities | 1.1 | 1-2 |
| - The program is easy to implement locally | 3.8 | 2-7 |
| - Low threshold for participation of inactive people | 3.9 | 1-7 |
| - The program matches the needs, wishes and possibilities of sports clubs | 5.2 | 2-11 |
| - **The program matches the NSF’s policy** | 5.5 | 2-11 |
| - The program is fully developed; it is of good quality and ready to be implemented | 6.8 | 4-10 |
| - **The NSF uses existing knowledge and experiences when developing the program** | 7.3 | 3-10 |
| - **The NSF collaborates with others when developing the program** | 7.6 | 2-12 |
| - The (implementation of the) program (locally) is not costly | 7.8 | 3-12 |
| - **The NSF runs pilot projects to test whether the program needs any adjustments** | 9.1 | 4-12 |
| - The name of the program is appealing to the target group and sports clubs | 9.9 | 7-12 |
| - The program is offered in the same way everywhere | 10.0 | 6-12 |

Table 2: Phase 1 Program development: impeding factors (n=6)

| **Impeding factor** | **Mean ranking score** | **Range assigned rankings** |
| --- | --- | --- |
| - The program does not match the target group’s needs, wishes and/or possibilities | 1.8 | 1-6 |
| - The (implementation of the) program (locally) is costly | 3.2 | 1-5 |
| - The program does not match the needs, wishes and/or possibilities of sports clubs | 3.3 | 1-5 |
| - The program is not easy to implement locally | 3.8 | 2-6 |
| - **The program (or parts of the program) is (are) not fully developed; it is not ready to be implemented** | 4.0 | 2-6 |
| - The program consists of one (training) session or a one day event; in this way it is more difficult to capture participants’ interest for the sport/a sports club membership | 4.8 | 1-6 |

Table 3: Phase 2 Organizational (pre)conditions: facilitating factors (n=10)

| **Facilitating factor** | **Mean ranking score** | **Range assigned rankings** |
| --- | --- | --- |
| - Having a “dedicated” program coordinator | 2.9 | 1-8 |
| - Sufficient time (in man-hours) to coordinate the program | 3.1 | 1-6 |
| - **Internal support for the program** | 3.9 | 1-9 |
| - Sufficient finances to coordinate and implement the program | 4.1 | 1-9 |
| - A good organizational structure for the program internally; tasks and responsibilities are properly assigned and clearly defined | 5.6 | 2-9 |
| - **External support for the program** | 5.8 | 2-10 |
| - Good external communication of the program | 6.9 | 4-9 |
| - Availability of trainer education and certification courses | 7.4 | 4-10 |
| - Good internal communication of the program | 7.5 | 4-10 |
| - **Availability of implementation materials** | 7.8 | 3-10 |

Table 4: Phase 2 Organizational (pre)conditions: impeding factors (n=3)

| **Impeding factor** | **Mean ranking score** | **Range assigned rankings** |
| --- | --- | --- |
| - Insufficient finances to coordinate and implement the program | 1.4 | 1-2 |
| - No or insufficient support for the program internally | 1.9 | 1-3 |
| - Internal organizational changes | 2.7 | 1-3 |

Table 5: Phase 3 Recruiting local sports clubs: facilitating factors (n=10)

| **Facilitating factor** | **Mean ranking score** | **Range assigned rankings** |
| --- | --- | --- |
| - Providing a complete (readily usable) package to sports clubs | 3.8 | 1-7 |
| - Approaching sports clubs personally | 4.3 | 1-10 |
| - Support for the program by sports clubs | 4.7 | 1-10 |
| - A good promotion/marketing strategy | 4.8 | 1-8 |
| - Providing financial support to sports clubs | 4.8 | 1-10 |
| - **Using a segmentation-based approach for recruiting sports clubs, based on expectations of successfulness** | 5.3 | 1-9 |
| - Sufficient qualified trainers locally | 5.3 | 1-10 |
| - Evidence of effectiveness and/or benefits of the program can be provided to sports clubs | 6.5 | 2-10 |
| - Collaboration with others to promote the program and/or recruit sports clubs | 7.4 | 4-10 |
| - Absence of competing programs/sports | 7.9 | 3-10 |

Table 6: Phase 3 Recruiting local sports clubs: impeding factors (n=6)

| **Impeding factor** | **Mean ranking score** | **Range assigned rankings** |
| --- | --- | --- |
| - No or insufficient qualified trainers locally | 2.2 | 1-4 |
| - No or insufficient support for the program by sports clubs | 2.4 | 1-6 |
| - Unavailability of additional (local) funding possibilities | 3.6 | 1-6 |
| - No (proper) sports accommodation, location and/or facilities locally | 3.9 | 1-6 |
| - No collaboration with others to promote the program and/or recruit sports clubs | 4.2 | 2-6 |
| - Presence of competing programs/sports | 4.8 | 1-6 |

Table 7: Phase 4 Recruiting participants: facilitating factors (n=9)

| **Facilitating factor** | **Mean ranking score** | **Range assigned rankings** |
| --- | --- | --- |
| - Support for the program by the target group | 3.3 | 1-6 |
| - A good promotion/marketing strategy nationally and locally | 3.4 | 1-8 |
| - The sports activities are organized in close proximity to the target group | 3.6 | 1-6 |
| - Sports clubs collaborate with other (local) parties to recruit participants | 4.1 | 1-9 |
| - Support of local promotion/marketing strategies by the NSF through provision of promotional materials and/or financial incentives | 4.1 | 2-8 |
| - The NSF collaborates with others to recruit participants | 5.1 | 2-8 |
| - The program reaches/engages inactive people | 6.2 | 2-9 |
| - Provision of sports equipment by the NSF or sports clubs at no (borrowing equipment) or low (hiring equipment) costs to facilitate participation | 7.3 | 3-9 |
| - Absence of competing programs or sports | 7.9 | 5-9 |

Table 8: Phase 4 Recruiting participants: impeding factors (n=7)

| **Impeding factor** | **Mean ranking score** | **Range assigned rankings** |
| --- | --- | --- |
| - The target group is unfamiliar with the program or the sport | 3.0 | 1-5 |
| - No or insufficient support for the program by the target group | 3.3 | 1-7 |
| - The program does not reach/engage inactive people | 3.4 | 1-5 |
| - The sports activities are not organized in close proximity to the target group | 3.7 | 1-6 |
| - Promotional/marketing materials and/or channels are not appropriate to the target group | 4.2 | 1-7 |
| - **Participation in the program is costly** | 4.4 | 2-7 |
| - Presence of competing programs or sports | 6.0 | 1-7 |

Table 9: Phase 5 Local implementation: facilitating factors (n=8)

| **Facilitating factor** | **Mean ranking score** | **Range assigned rankings** |
| --- | --- | --- |
| - Enthusiastic people within sports clubs delivering (high-) quality performances | 2.9 | 1-7 |
| - Sports clubs are (personally) supported by the NSF when implementing the program locally | 3.8 | 1-7 |
| - Availability of follow-up sports activities locally that match participants’ needs, wishes and possibilities | 3.8 | 1-8 |
| - Personal/good communication between the NSF and sports clubs | 4.0 | 1-8 |
| - Enthusiastic participants; they stimulate both the sports clubs (to continue their efforts) as well as other participants | 4.2 | 1-8 |
| - **A good organizational structure for the program locally; tasks and responsibilities are properly assigned and clearly defined** | 4.8 | 1-7 |
| - Opportunities for sports clubs (provided by the NSF) to exchange knowledge and experiences | 5.9 | 2-8 |
| - Sports clubs collaborate with other (local) parties to run the program locally | 6.7 | 2-8 |

Table 10: Phase 5 Local implementation: impeding factors (n=5)

| **Impeding factor** | **Mean ranking score** | **Range assigned rankings** |
| --- | --- | --- |
| - No enthusiastic and/or incompetent people within sports clubs | 1.5 | 1-4 |
| - No clear division of roles, tasks and responsibilities between the NSF and sports clubs | 3.0 | 1-5 |
| - No (appropriate) follow-up sports activities for participants locally | 3.2 | 1-5 |
| - Insufficient personal contact between the NSF and sports clubs | 3.3 | 2-5 |
| - Sports clubs do not comply with the NSF’s implementation instructions and/or rules | 4.0 | 1-5 |

Table 11: Phase 6 Securing continuation of the program: facilitating factors (n=7)

| **Facilitating factor** | **Mean ranking score** | **Range assigned rankings** |
| --- | --- | --- |
| - **The program is part of the NSF’s long-term policy** | 2.6 | 1-5 |
| - **The NSF has sufficient financial resources available to continue the program/secure the program for the future** | 2.7 | 1-6 |
| - **The program is part of the sports club’s long-term policy** | 3.0 | 1-6 |
| - **Sports clubs have sufficient financial resources available to continue the program locally/secure the program for the future** | 3.8 | 1-7 |
| - **Evaluation of the program (and/or the implementation process) to identify necessary adjustments** | 4.8 | 1-7 |
| - **The NSF collaborates with others to continue the program/secure the program for the future** | 5.5 | 2-7 |
| - **Sports clubs collaborate with others to continue the program/secure the program for the future** | 5.7 | 2-7 |

Table 12: Phase 6 Securing continuation of the program: impeding factors (n=2)

| **Impeding factor** | **Mean ranking score** | **Range assigned rankings** |
| --- | --- | --- |
| - The NSF has insufficient financial resources available to continue the program/secure the program for the future | 1.4 | 1-2 |
| - **Sports clubs have insufficient financial resources available to continue the program locally/secure the program for the future** | 1.6 | 1-2 |

1. Mean ranking score: For each factor: Sum of rankings divided by the number of program coordinators (n=12); Range assigned rankings: Lowest and highest ranking of factor. [↑](#footnote-ref-1)
